# mRNA-based immunotherapy platform targeting endometrial cancer

**DOI:** 10.1186/s12967-025-07497-w

**Published:** 2025-12-05

**Authors:** Moritz Freyberg, Martha Dierks, Norbert Nass, Christopher George, Maria Geffken, Atanas Ignatov, Thomas Kalinski, Zoya Ignatova

**Affiliations:** 1 Biochemistry and Molecular Biology, University of Hamburg, Hamburg, Germany; 2 Institute of Pathology, University Hospital Brandenburg an der Havel Brandenburg and Medical School Theodor Fontane (MHB), Brandenburg, Germany; 3 Department of Transfusion Medicine, University Medical Center Hamburg-Eppendorf, Hamburg, Germany; 4 Department of Gynecology and Obstetrics, Otto-von-Guericke University, Magdeburg, Germany

## Abstract

**Background:**

Conventional therapies for endometrial cancer (EC), one of the most prevalent gynecological malignancies, remain inefficient, particularly in advanced stages and relapse with chemoresistant disease, underscoring the urgent need for new therapeutic strategies. Here, we present an approach and key considerations for developing a safe ex vivo mRNA-based vaccine potentially targeting EC.

**Methods:**

Based on expression analysis in patient-derived endometrial tumor tissues, we identify two potential targets – fibroblast activation protein (FAP) and melanoma-associated antigen A4 (MAGEA4) – for immunotherapy using an ex vivo vaccine setting. We optimized the individual components of the mRNA expression cassette to enhance translation fidelity and antigen expression.

**Results:**

We selected an optimal 5′ untranslated region (UTR) and fine-tuned the translation termination signal to prevent readthrough and generation of unintended neoantigens. Functionally, the ex vivo mRNA vaccine elicited a robust T cell response, particularly when combining both FAP and MAGEA4 antigens. MAGEA4 expression correlated with disease severity in EC tumor tissue, reinforcing its relevance as both a prognostic marker and immunotherapeutic target. Its combination with FAP, expressed by both EC cells and cancer-associated fibroblasts, as part of the tumor microenvironment, may facilitate immune evasion and promote tumor infiltration.

**Conclusions:**

Our data provides evidence that FAP and MAGEA4 antigens delivered via mRNA can drive effective T cell activation. This proof-of-concept study establishes a framework for developing immune-based interventions and more effective treatment strategies for patients with EC.

**Supplementary Information:**

The online version contains supplementary material available at 10.1186/s12967-025-07497-w.

## Introduction

Endometrial cancer (EC) is a malignant tumor of the inner lining of the uterus, with a constantly increasing incidence and mortality rate [1]. It ranks among the ten most common malignant tumors in women worldwide (e.g. with an annual incidence of approximately 11.000 new cases in Germany) and the most frequent pelvic malignancy in women [2]. A variety of risk and prognostic factors contribute to the pathogenesis of EC and significantly impact on patient survival [1]. In addition to the patient’s age, the extent of disease dissemination is a key determinant of disease staging and prognosis according to FIGO [3]. Despite established conventional treatments, e.g. surgery, chemotherapy, and radiotherapy, frequent chemoresistance and relapse still poses a significant challenge for tumor treatment, particularly at advanced stages of disease [4, 5].

Recently, cancer immunotherapy, which leverages the immune system to deactivate malignant cells, has gained considerable attention as a potential therapy for EC. In particular, immune checkpoint blockade targeting the PD1/PD-L1 axis has demonstrated promising results in a subset of EC patients [6]. Tumors with mismatch repair deficiency (dMMR) or high microsatellite instability (MSI-H) are especially immunogenic. Pembrolizumab and dostarlimab are clinically approved for the treatment of recurrent EC in patients with dMMR. In this EC subgroup, this anti-PD1 therapy achieves response rates of 40–45% [6, 7]. However, the majority of endometrial tumors are microsatellite-stable and respond poorly to checkpoint inhibitors [8–10]. Not all dMMR tumors respond [6]. Together, these limitations of current treatments underscore the need for complementary immune strategies and have raised interest in active immunotherapies that can prime tumor-specific T cells *de novo*, such as therapeutic cancer vaccines [11]. Currently, immunotherapy in EC has predominantly focused on Wilms tumor protein encoded by *WT1* as a potential target antigen [12], which is overexpressed in up to 79% of ECs [13].

Among different vaccine platforms, mRNA vaccines have emerged as an auspicious approach in oncology. Recent clinical studies in solid tumors (pancreatic and melanoma) have shown that mRNA vaccines can induce robust antigen-specific T cell responses and prolong disease-free survival [14, 15]. Despite these promising results, challenges such as low mRNA expression levels and the potential generation of undesired neoepitopes [16, 17] remain significant obstacles in mRNA vaccine platforms. Two main strategies for mRNA-based vaccines, namely in vivo and ex vivo vaccines, have been pursued [18]. For the in vivo vaccine, a modified mRNA is delivered directly to the patient to drive antigen production and prime the T cells for the desired antigen. Several candidates utilizing the in vivo approach are currently undergoing clinical evaluation, including vaccines targeting non-small cell lung cancer and melanoma (NCT04526899, NCT06077760). In in vivo vaccine settings, delivery must overcome the challenge of targeting mRNA to antigen-presenting cells (APCs), to avoid undesired biodistribution and degradation in the body [19]. Autologous cells are engineered in vitro to express mRNA encoding the antigen of interest and then administered as a cellular vaccine. Thus, ex vivo platforms allow for tight control of antigen delivery to the immune system [20]. mRNA is introduced into dendritic cells (DCs) or other immune cells ex vivo, selecting only for APCs carrying the tumor antigens. The modified cells can be matured and optimized, leading to more efficient priming of tumor-specific T cells [21, 22]. Several ex vivo vaccines are currently under clinical evaluation, including vaccines targeting myeloid leukemia and gastric tumors (NCT01686334, NCT03185429). To date, no mRNA vaccines targeting EC have advanced to clinical evaluation.

Here, we report the development and evaluation of an ex vivo mRNA vaccine platform targeting EC. Expression analysis from the Protein Data Atlas and immunohistochemical analysis of surgically removed tumors from EC patients were used to select two antigens, e.g. melanoma-associated antigen 4 (MAGEA4*)* and fibroblast activation protein (FAP), which correlate with patients’ survival and are thus suitable targets for mRNA-based immunotherapy of EC. We optimized the mRNA expression cassette to achieve efficient T cell activation and enhance the safety of the vaccine. Our results establish the foundation for an ex vivo mRNA vaccine and immunotherapeutic treatment of EC, which could be integrated with current therapies to improve clinical outcomes.

## Methods

### Immunohistochemistry of patients’ tissues

In this retrospective study, we re-analyzed archived samples from primary EC tumor tissue of 209 patients diagnosed with EC between 2005 and 2020 at the Department of Gynecology in the Clinic Brandenburg an der Havel. The primary tumor tissue was obtained during the initial diagnosis and archived and stored at the Department of Pathology (University Hospital Brandenburg an der Havel, Brandenburg, Germany). The ethics approval (9082022-BO-E-RETRO) for this study has been obtained from the Research and Ethics Committee of the University Hospital Brandenburg an der Havel (Brandenburg, Germany). Patients gave written informed consent before surgery. Additional individual consent for this re-analysis post-surgery was not needed.

The archived primary EC tumor material of 209 patients were newly immunohistochemically stained and analyzed within this study for expression of FAP, MAGEA4, CT83 and CEP55 and compared with clinical outcomes, including histological subtype, FIGO classification, disease-free survival DFS, and OS. The follow-up period was set at five years.

Specific primary mouse and rabbit antibodies were used to detect and bind the target antigens in formalin-fixed, paraffin-embedded (FFPE) tissue sections. Monoclonal antibodies directed against FAP (FAP/4854; 1:100 dilution; #ZL4561943) and MAGEA4 (CPTC-MAGEA4-1; 1:1000; #ZL4561944) were both obtained from Thermo Fisher Scientific. Antibodies were diluted in antibody dilution buffer (Vetana). Staining was performed on a Ventana Benchmark system. After deparaffinization, antigen demasking was achieved by heating at 95 °C in buffer CC1 (Ventana). Signal amplification and detection were done using the Ventana OptiView Amplification Kit and the OptiView DAB IHC Detection Kit. After staining, slides were counterstained using hematoxylin (Bluing Reagent) and then covered. For double staining, FAP was combined with an anti-CD45 monoclonal rabbit antibody (1:100 dilution; Cell Signaling Technology, #13917). The demasking procedure was performed on a Leica system using the Leica ER2-demasking buffer. Detection was achieved with the Chromoplex 1 Dual Detection Kit (Leica).

For CT83, all tested samples yielded negative results. CEP55 could not be detected using the commercially available antibodies. Some samples could not be stained and were excluded. In total, 192 samples for MAGEA4 and 205 samples for FAP were successfully processed and considered in the analysis.

Immune reactivity scores (IRS) were calculated using on the Remmele score [23] and are derived by multiplying the percentage of stained cells (P) by staining intensity (I) (IRS = P x I). The scoring for the quantitative assessment in percentage terms was assigned as follows: 0 – for 0% stained cells in the entire tissue section, 1 – for < 10% stained cells, 2 – for 10–50% stained cells, 3 – for 51–80% stained cells, and 4 – for >80% stained cells in the entire tissue section. The staining intensity was assessed using a scale from 0 to 3.

### mRNA design

The DNA template used to produce the mRNA in the in vitro transcription (IVT) reaction was constructed from different modules (Supplementary Table 2). The polyA tail, linkers and 5’UTR sequences were purchased as oligonucleotides (Microsynth, Switzerland). The remaining parts of the mRNA were amplified using the total cDNA of HeLa cells or PBMCs. The individual parts of the DNA template were connected by overlap PCR. Thereafter, the 5’ ends were extended by a T7 promoter sequence (5’ TAATACGACTCACTATAAG 3’) using PCR. The final DNA template sequences were cloned into a commercially available vector (pGL4.51 [luc2 CMV Neo]; Promega, Germany).

To assess the effects of stop codon identity on readthrough, different stop codons and flanking sequences were introduced using oligonucleotides purchased from Microsynth (Switzerland). These oligonucleotides consisted of either the stop codons UGA(C), UAA(C), UAA(A), UAAUAA(C) or the sense GGA codon flanked by 33 bp upstream and 3 bp downstream of the designed mRNA sequence, i.e. from MITD (upstream) and AES (downstream). Oligonucleotides were amplified by PCR and cloned into the pSGDlucV 3.0 vector (Addgene, USA) for IVT. The resulting sequences will be referred to as readthrough reporter mRNAs.

### In vitro transcription (IVT) of mRNA

DNA plasmids, serving as templates in the IVT reaction for all experiments except stop codon readthrough assessment, were digested with *Sap*I (New England Biolabs). Modification of the T7 promoter to harbor the initiating bases AG instead of GN allowed for co-transcriptional capping with the CleanCap reagent. Consequently, plasmids were subjected to IVT using HiScribe T7 mRNA Kit with CleanCap Reagent AG (New England Biolabs) following the manufacturer’s protocol. If not stated otherwise, all uridines were replaced by m^1^Ψ (Jena Bioscience).

DNA plasmids, serving as templates in the IVT reaction, for readthrough reporter mRNA were digested with *Xba*I (Thermo Fisher Scientific). These plasmids were commercially acquired and, therefore their T7 promoter was not further modified for use with the CleanCap reagent. These plasmids were subjected to IVT using the HighYield T7 ARCA mRNA Synthesis Kit (Jena Bioscience) following the manufacturer’s protocol. For the respective mRNAs, uridines were replaced by m^1^Ψ, Ψ or 5-moU (Jena Bioscience).

### Cell lines

EL4 cells (TIB-3) were acquired from ATCC and cultured in RPMI 1640 medium (Pan Biotech), containing 10% FBS and 2mM L-glutamine (Thermo Fisher Scientific). HEK293XL-hTLR7/hTLR8 cell lines were purchased from InvivoGen and cultured in DMEM medium (PAN-Biotech), containing 10% FBS and 2mM L-glutamine.

### PBMC isolation

PBMCs were obtained from buffy coats from individual healthy blood donors, who had donated blood in the period 2022–2024 at the University Hospital Hamburg, Germany. Leftovers of samples not used for clinical purposes were used for research; the individuals gave written informed consent before blood donation. PBMCs were isolated using the Ficoll-Paque PLUS density gradient method. Briefly, buffy coats were diluted with equal amounts of 1x PBS and layered on top of Ficoll-Paque PLUS density gradient media (Cytiva). After centrifugation, the PBMC layer was transferred into a new conical tube and washed four times with 1x PBS. PBMCs were counted and flash frozen in 10% heat-inactivated FBS and 10% DMSO (Sigma Aldrich) until further use.

### Monocyte isolation and differentiation to immature modcs

Monocytes were isolated from PBMCs utilizing human CD14 MicroBeads (Miltenyi Biotec) following the manufacturer’s protocol. The monocytes were cultured in RPMI 1640 medium (Pan Biotech), supplemented with 10% heat-inactivated FBS and 2 mM L-glutamine. For differentiation into immature moDCs, IL-4 (250 U/ml, Miltenyi Biotec) and GM-CSF (1000 U/ml, Miltenyi Biotec) were added directly to the cell culture media. After 48 h, cells were re-stimulated by repeated addition of IL-4 and GM-CSF. On day 5, the immature moDCs were harvested for further experiments.

### CD8 + T cell isolation

CD8 + T cells were isolated from PBMCs utilizing the human CD8 + T cell Isolation Kit (Miltenyi Biotec) following the manufacturer’s protocol. Isolated T cells were cultured in RPMI 1640 medium (Pan Biotech), supplemented with 10% heat-inactivated FBS and 2 mM L-glutamine overnight. On the next day, CD8 + T cells were harvested for downstream experiments.

### Dual-Luciferase assay

Immature moDCs were seeded in alcian blue-coated 96-well cell culture plates. After 24 h, cells were transfected with the corresponding mRNA using Lipofectamine MessengerMax (Thermo Fisher Scientific) according to the manufacturer’s protocol.

To assess the impact of the 5’UTR on protein expression, cells were transfected with mRNA (100 ng) encoding *RLuc*, which contained various 5’UTRs. A commercially available *FLuc* mRNA (TriLink Biotechnologies) was co-transfected for normalization.

For stop codon readthrough, cells were transfected with the respective reporter mRNAs (100 ng) harboring either UGA(C), UAA(C), UAA(A), UAAUAA(C) stop codons or GGA codon and IVT synthesized using different uridine analogs (m^1^Ψ, Ψ or 5moU; Jena Bioscience). After 24 h, cells were lysed with 1x passive lysis buffer and fluorescence was determined using the Dual-Luciferase reporter assay system (Promega). Protein expression levels were determined by the RLuc/Fluc ratio. The percentage of readthrough was calculated by normalizing to the RLuc/Fluc ratio of the construct harboring the sense GGA codon.

### mRNA immunogenicity assessment

HEK293XL-hTLR7, HEK293XL-hTLR8 cells or immature moDCs were seeded in 96-well culture plates. After 24 h, cells were transfected with *RLuc*-encoding designed mRNAs using protamine, a commonly used reagent to test the immunostimulatory effect of RNAs [24]. Resiquimod (R848, InvivoGen), a TLR7/8 antagonist, or OD-2216 (InvivoGen), a TLR9 antagonist, served as positive controls for TLR activation [25]. R848, OD-2216 and mRNA were diluted to 0.5 µg/µl and incubated with equal amounts of protamine for 10 min at room temperature. Subsequently, 5 µg/ml of the mRNA protamine mix and 1 µg/ml of the R848 or OD-2216 protamine mix were added directly to the cells. After 1 h, the medium was replaced with fresh medium and after 7 h the supernatants were collected by centrifugation (10 min at 300xg). The levels of IL-8, TNF-α, and IFN-γ levels were then measured using ELISA Flex kit (Mabtech).

### MoDCs maturation analysis

Immature moDCs were seeded in a 6-well microtiter plate and using Lipofectamine MessengerMax (Thermo Fisher Scientifc) were transfected with *RLuc*-encoding designed mRNAs (2.5 µg) containing different ratios of U: m^1^Ψ (100:0; 50:50 and 0:100). As a positive control, immature moDCs were treated with a maturation cocktail consisting of IL-1β (13.2 ng/ml, Sigma Aldrich), IL-6 (150 ng/ml, Sigma Aldrich), TNFα (10 ng/ml, Biomol) and Prostaglandin E2 (1 µg/ml, Biomol). After 24 h, the cells were stained for flow cytometry with APC-conjugated anti-human HLA-DR and FITC-conjugated anti-human CD86 antibodies (Miltenyi Biotech). Stained cells were subsequently subjected to flow cytometry.

### Antigen-presentation assay

The epitope presentation levels were measured by transfecting EL4 cells with SIINFEKL-encoding designed mRNA (2.5 µg) using Lipofectamine MessengerMax (Thermo Fisher Scientific). After 24 h, the cells were stained for flow cytometry with an APC-conjugated anti-mouse H-2Kb/SIINFEKL antibody (Miltenyi Biotech) and subjected to flow cytometry using FACSCalibur (BD Biosciences).

### T-cell activation and proliferation

Immature moDCs were seeded in 6-well plates and transfected with encoding MAGEA4 or FAP (produced by IVT, with all uridines were replaced by m^1^Ψ) using Lipofectamine MessengerMax (Thermo Fisher Scientific). One hour after transfection, cells were matured with a maturation cocktail containing IL-1β (13.2 ng/ml, Sigma Aldrich), IL-6 (150 ng/ml, Sigma Aldrich), TNFα (10 ng/ml, Biomol), and Prostaglandin E2 (1 µg/ml, Biomol). After 24 h, moDCs were harvested and resuspended in ImmunoCult- XF T cell Expansion Medium.

CD8 + T cells isolated from the PBMCs of the same donor were resuspended in 1x PBS buffer (Thermo Fisher Scientific), supplemented with 5% heat-inactivated FBS, following labeling with 50 µM of carboxyfluorescein succinimidyl ester (CFSE) (Biomol) for 5 min at room temperature. Labeled CD8 + T cells were washed with 1x PBS, 5% heat-inactivated FBS, resuspended in ImmunoCult- XF T cell Expansion Medium and co-cultured with mature moDCS at a 10:1 ratio in 96-well U-bottom plates (Greiner Bio-One) to monitor proliferation and activation. After 5 days of co-culture, T-cell activation was analyzed using ELISA (ELISA Flex Kits, Mabtech) by measuring the levels of released IL-2, TNFα and IFNγ in the cell culture media. On the same day, proliferation was assessed by subjecting T cells to flow cytometry and measuring the CFSE intensity, whose fluorescence progressively decreased with each cell division. The reduced CFSE intensity is a readout of cell proliferation.

### Statistical analysis

Statistical analyses for the patient cohort were performed using SPSS version 22 (IBM). Cross-tabulation was applied to assess correlations between clinicopathological parameters and FAP or MAGEA4 positivity. Statistical significance was determined using Fisher’s exact test or Pearson’s Chi^2^ test, and correlations were analyzed using Spearman’s rank correlation coefficient. DFS and OS were evaluated using the Kaplan-Meier method, and differences in survival curves were assessed with the log-rank test. DFS was defined as the period between the diagnosis date and the date of local and/or regional recurrence, diagnosis of distant metastases, or death, whichever occurred first. The secondary outcome was the OS, which is defined as the time from the date of diagnosis to death of any cause. A univariate Cox proportional hazards regression model was used to identify significant prognostic factors.

## Results

### Selection of FAP and MAGEA4 as potential antigens for an ex vivo vaccine

To develop an ex vivo vaccine targeting EC, we first sought to identify potential antigen targets. We used the Human protein atlas [26], which contains expression data from RNA-seq and immunohistochemistry of tissue samples from a variety of tumors, including EC. We considered a multi-step selection procedure based on several criteria to filter for suitable candidates. In the initial round, we selected transcripts expressed in at least 15% of all endometrial tumor samples based on mRNA expression from RNA-seq data. The threshold of 15% was selected based on the typical expression range of cancer-testis antigens in tumors (e.g. 10–30%), which are considered suitable vaccine targets [27–29]. Choosing the lower end of this range allowed inclusion of low-expressed transcripts, thereby minimizing the exclusion of potentially relevant candidates while maintaining specificity by excluding very low-prevalence antigens. Subsequently, we considered the protein expression level in the immunohistochemical analysis in the Human protein atlas [26], using one of the following criteria: (i) exclusive expression in endometrial tumor cells; (ii) overexpression in EC compared to adult healthy tissue; or (iii) only expression in EC and adult testis tissue, as a tissue not present in females. At the final step, we stratified selection of the antigens based on published reports on the immunogenicity of the candidates. In this context, immunogenicity refers to the capacity of a tumor-associated protein to elicit adaptive immune responses, including antibody production and activation of CD4⁺ and CD8⁺ T cells in cancer patients. Proteins with established immunogenic properties were considered ideal candidates, because they are more likely to elicit a strong T-cell response. Using this multi-step selection process, we identified four candidate antigens for potential use in ex vivo mRNA vaccine setup targeting EC: melanoma-associated antigen 4 (MAGEA4) (UniProtKB P43358), fibroblast activation protein alpha (FAP) (UniProtKB Q12884), centrosomal protein 55 (CEP55) (UniProtKB Q53EZ4) and cancer/testis antigen 83 (CT83) (UniProtKB Q5H943).

To further assess their potential as antigen targets, we analyzed archived tissue samples from 209 patients with various EC stages that were collected at the Department of Gynecology in Brandenburg an der Havel between 2005 and 2020. We calculated immune reactivity scores (IRS) based on the Remmele score [23]. A density score was assigned as follows: 0 – for preparations with 0% stained cells across the entire tissue section; 1 – for < 10% stained cells; 2 points for 10–50% stained cells; 3 – for 51–80% stained cells; and 4 – for >80% stained cells. The staining intensity was assessed using a scale from 0 to 3 (i.e. no staining (0), weak (1), moderate (2) and vigorous (3) staining). For CT83, all tested samples yielded negative results. CEP55 could not be detected using commercially available antibodies. Consequently, both were excluded from subsequent analyses. Reliable staining was obtained from 192 samples for MAGEA4 and 205 samples for FAP expression. We used the median as a cut-off value, which was >7 for FAP and >0 for MAGEA4. For the MAGEA4, 45 out of 192 (23.4%) samples were classified as positive (Fig. S1). FAP expression was detectable in 106 out of 205 (51.7%) samples (Fig. S1). In total, 41 samples (21.3%) were double-positive for both FAP and MAGEA4 expression. Notably, while the MAGEA4 protein was exclusively detected in the tumor cells, FAP was observed in both tumor and surrounding stroma cells (Fig. S2 and S3).

### MAGEA4 expression correlates with disease severity

To further investigate the effect of MAGEA4 and FAP on EC, we analyzed the clinicopathological data (Supplementary Table 1). The tumors were analyzed by immunostaining and classified as FIGO >II. In EC, the FIGO stage higher than II indicates expansion of the cancer beyond the uterus. It involves either invasion of the adnexa (stage IIIA), vaginal or parametrial involvement (stage IIIB), pelvic or para-aortic lymph node metastases (stage IIIC), or distant metastases (stage IV) [30]. The MAGEA4 expression increased significantly with the following parameters: age (>70 years; *P* ≤ 0.001); advanced disease stage (FIGO >II; *P* = 0.001); lymph node involvement (*P* = 0.026), and tumor grading (*P* < 0.001) (Supplementary Table 1). Furthermore, MAGEA4 expression was markedly elevated in non-endometrioid EC cases (57.1%, *P* < 0.001) and correlated with increased mortality (*P* = 0.049) (Supplementary Table 1). In contrast, the FAP expression did not correlate with the clinicopathological parameters (Supplementary Table 1). Moreover, the FAP expression was comparable between endometrioid and non-endometrioid EC cases (49.1% vs. 38.2%) (Supplementary Table 1).

**Fig. 1:**
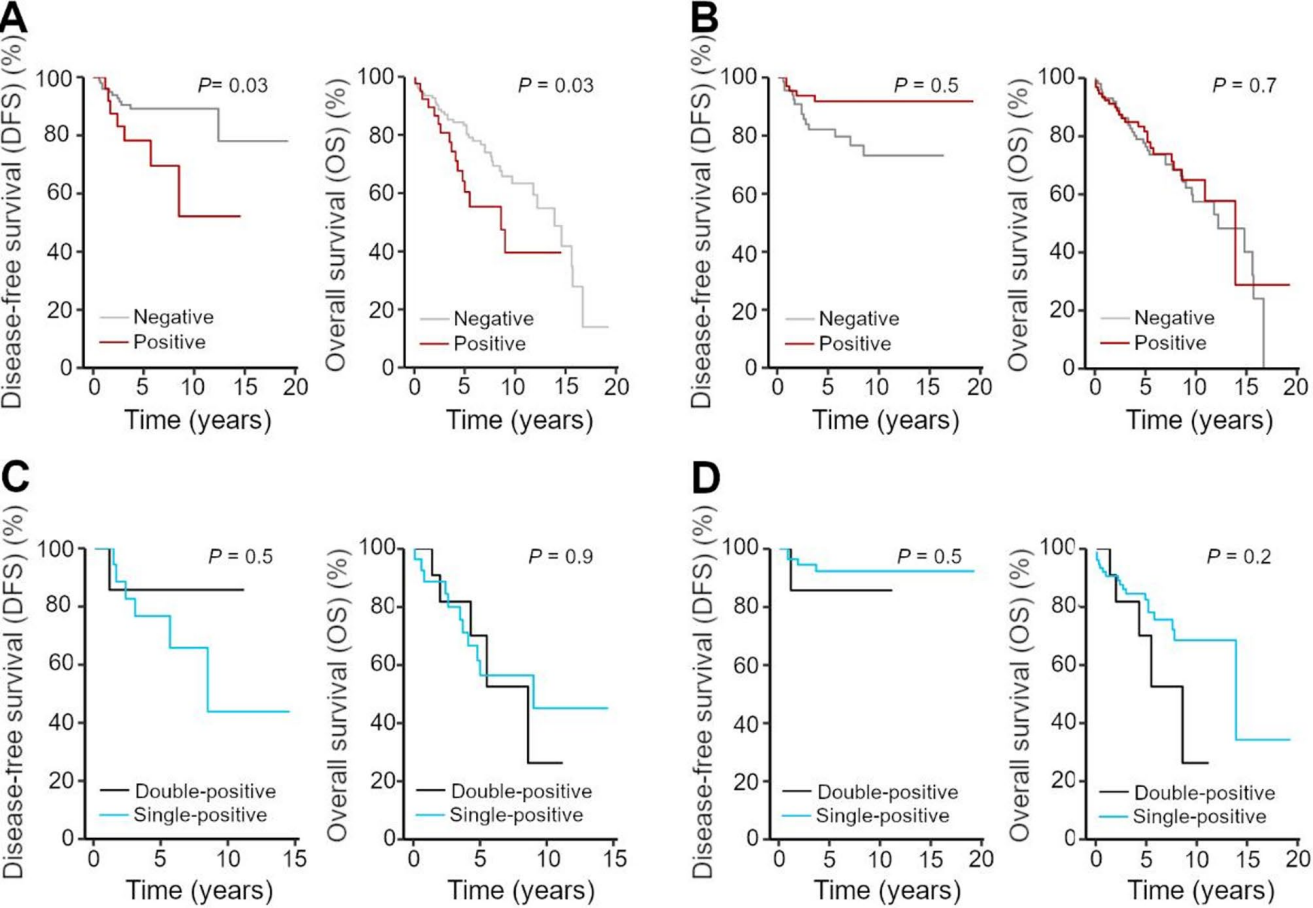
MAGEA4 expression correlates with DFS and OS. **A-D** Kaplan-Meier survival analyses of DFS and OS in MAGEA4-positive (red) vs. MAGEA4-negative (grey) (panel A), FAP-positive (red) vs. FAP-negative (grey) (panel B), double-positive (black) vs. MAGEA4-positive (blue) (panel C), and double-positive (black) vs. FAP-positive (blue) (panel D)

Kaplan-Meier analyses assessing the prognostic relevance of FAP and MAGEA4 in EC revealed distinct patterns regarding disease-free survival (DFS) and overall survival (OS) (Fig. 1). For MAGEA4, we observed a pronounced separation in DFS and MAGEA4-positive patients experienced much earlier disease recurrences (*P* = 0.03) (Fig. 1A). Regarding OS, we observed an increased mortality rate and earlier occurrence of tumor-related events (recurrence or death) in MAGEA4-positive EC with longer follow-up (*P* = 0.03) (Fig. 1A). Thereby, the negative impact on long-term prognosis was reflected in both earlier recurrence development and increased overall mortality. For FAP, we observed a trend toward poorer DFS in FAP-negative patients (Fig. 1B). In contrast, no significant difference in OS was detected between FAP-positive and FAP-negative patients (Fig. 1B).

Comparison between single-positive (i.e. expressing FAP or MAGEA4 alone) and double-positive (i.e. expressing both FAP and MAGEA4) patient subgroups did not reveal significant differences in survival outcomes (Fig. 1C, D). Specifically, we observed no significant differences in the OS (*P* = 0.2) or DFS (*P* = 0.5) between FAP-positive and double-positive (FAP and MAGEA4) patients (Fig. 1D). Similarly, the comparison between MAGEA4-positive and double-positive patients showed no significant differences in OS (*P* = 0.9) or DFS (*P* = 0.5) (Fig. 1C). Notably, the number of double-positive cases was markedly lower (14 and 10 patients, respectively) than in the single-positive cohorts, thereby limiting statistical power.

The univariable Cox regression identified several significant predictors of DFS and OS in patients with EC (Table 1). Regarding DFS, patients with MAGEA4-positive tumors exhibited ~ 3-fold higher risk of tumor recurrence (HR = 2.884, Table 1). Lymph node involvement was a stronger predictor, associated with a nearly 4-fold higher risk of recurrence (HR = 4.291; Table 1). The FIGO stage showed the most pronounced effect, with patients in advanced stages exhibiting an 8-fold higher risk of tumor recurrence (HR = 8.344; Table 1). In contrast, FAP expression showed a trend towards a protective effect (HR = 0.352), although the statistical significance was only narrowly reached (*P* = 0.045; Table 1). For OS, MAGEA4 expression was also associated with ~ 2-fold higher risk of mortality (HR = 1.908; Table 1). Additionally, older patients had a significantly higher mortality risk (HR = 1.764), while lymph node involvement was linked to a higher risk of disease-related death (HR = 3.512; Table 1). The FIGO staging remained the strongest prognostic factor. Patients in advanced stages (FIGO III/IV) exhibited 3- to 4-fold increased mortality risk (HR = 3.514; Table 1). Together, these data demonstrate that while both MAGEA4 and FAP are expressed in EC, only MAGEA4 correlates with disease severity.

**Table 1:** Univariable survival analysis. A Cox regression model was used based on specific clinicopathological data. Hazard ratio (HR) for DFS is measured from the time of initial diagnosis to recurrence or last known follow-up. For OS, it reflects the risk of EC-related death from diagnosis to the last known follow-up. CI, confidence interval

Parameter	DFS			OS		
	HR	95% CI	*P* value	HR	95% CI	*P* value
MAGEA4	2.884	1.098–7.370	0.031	1.908	1.057–3.447	0.032
FAP	0.352	0.127–0.979	0.045	0.897	0.533–1.511	0.683
Age > 70	2.210	0.868–5.627	0.096	1.764	1.348–2.308	< 0.001
Grading	1.371	0.491–3.824	0.547	1.097	0.590–2.040	0.770
N-Status	4.291	1.246–14.775	0.021	3.512	1.381–8.931	0.008
FIGO > II	8.344	2.668–26.095	< 0.001	3.514	1.551–7.960	0.003

### Design of the key elements of an ex vivo vaccine

**Fig. 2:**
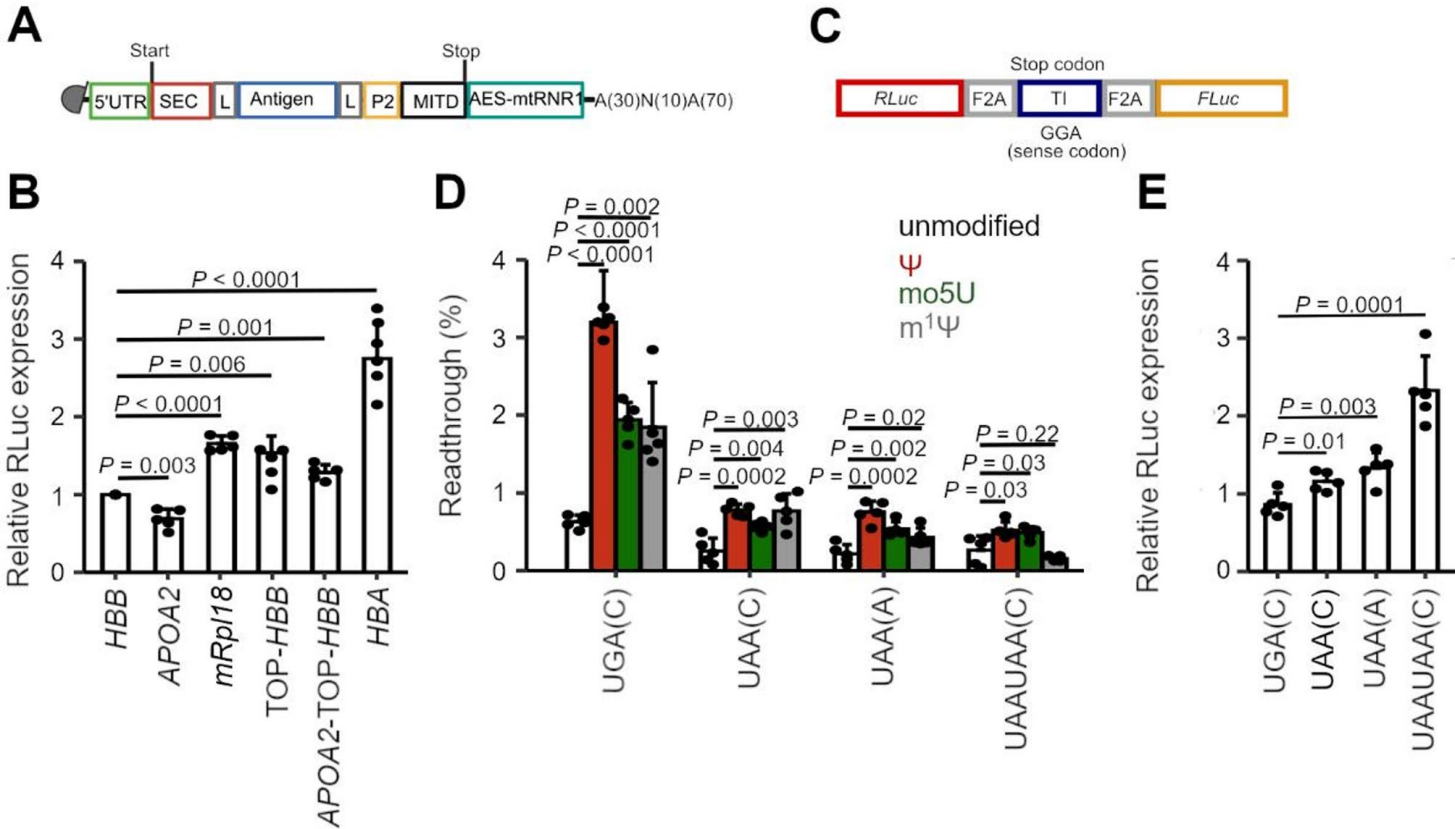
mRNA vaccine design and optimization of the 5’UTRs and termination sequence. **A** mRNA design for EC vaccine; SEC, MHC class I secretion signal; P2, Tetanus toxoid CD4 + T cell epitope; MITD = MHC class I trafficking signal; L, protein linker; AES-mtRNR1; hybrid 5’UTR sequence of amino-terminal enhancer of split and mitochondrially encoded 12S ribosomal RNA. **B** Effect of various 5’ UTRs on the expression of RLuc reporter normalized to FLuc in moDCs transfected with IVT mRNAs. *HBB*, human β-globin; *HBA*, human α-globin. **C** Reporter mRNA design for assessing the stop codon readthrough. The test insert (TI) consists of 33 base pairs (bp) upstream and 3 bp downstream of the stop codon. Replacement of a stop codon by the sense GGA codon serves as a positive control. *RLuc* and *FLuc* denote Renilla luciferase and Firefly luciferase sequences, respectively. F2A sequences mediate co-translational ‘self-cleavage’ between individual parts, avoiding interference from protein fusion. **D** Readthrough level at different stop codons assessed in moDCs 24 h after transfection with modified IVT mRNA. Expression of the stop codon-containing mRNAs was normalized to that of the sense GGA codon. Nucleotides in brackets represent the + 4 nucleotide. **E** Relative expression of *RLuc* mRNA (panel A) in moDC 24 h post-transfection with IVT mRNA modified with m^1^Ψ. The expression was normalized to that of FLuc. Data in **B**, **D** and **E** are means ± SD (*n* = 5 biological replicates). P values were calculated by a two-tailed, unpaired t-test

Having identified MAGEA4 and FAP as potential antigen targets, we next designed the expression cassette of the mRNA vaccine. We focused on an ex vivo mRNA vaccine, which, compared to the circulating mRNA vaccine approach, offers two key advantages: (i) the mRNA delivery into cells drastically increases efficiency and eliminates off-target delivery and biodistribution [18], and (ii) in vitro priming of T cells allows for introducing only activated T cells. Considering recent developments in vaccine research, we identified several elements in the expression cassette (Supplementary Table 2) that have been commonly used in mRNA-based vaccines targeting different cancers [31–34]. Along with the antigen sequence, mRNA cassettes typically incorporate additional elements to enhance epitope processing and presentation, a relatively inefficient process [35]. Among these elements are MHC class I secretion signal (SEC), an MHC class I trafficking signal (MITD), and the Tetanus toxoid CD4 + T cell epitope P2 [36, 37]. We incorporated these three elements into an mRNA cassette (Fig. 2A) and connected them with the antigen sequence via flexible linkers encoding two consecutive amino acid stretches (GGSGG; Fig. 2A), facilitating the antigen processing and presentation.

Systematic analysis of the effect of different 3’UTR sequences on expression in DCs highlights one hybrid 3’UTR [38] that outperforms commonly used 3’UTRs in other mRNA vaccines [32, 39, 40]. We included the best performing 3’UTR (Fig. 2A), which is a hybrid sequence of the amino-terminal enhancer of split (AES) and the mitochondrially encoded 12S rRNA (mtRNR1). The polyA tail plays a crucial role in mRNA stability and translation efficiency, yet it poses a significant challenge during cloning and plasmid amplification in bacteria. We used segmented polyA (Fig. 2A), as previously established [41]. The polyA region consists of 100 adenosines and is interrupted by a 10-nucleotide-long stretch (5’TGCATGCACG3’). Compared to a polyA tail with consecutive adenosines, this segmented polyA stretch markedly reduced DNA plasmid recombination, while not compromising translation or mRNA half-life [42].

### Optimization of the 5’UTR for maximal expression

The 5’UTR is the most critical element affecting expression by modulating both ribosomal loading and mRNA stability [43]. We next investigated the effect of various 5’UTRs in monocyte-derived dendritic cells (moDCs). We compared the expression to that driven by the 5’UTR of β-globin (*HBB)* – a commonly used 5’UTR in mRNA vaccines [44]. We selected the four 5’UTRs from natural genes: apolipoprotein A-II (*APOA2)*, terminal oligopyrimidine motif β-globin *(TOP-HBB)*, mitochondrial ribosomal protein L18 (*mRPL18)*, and α-globin (*HBA*) (Supplementary Table 3). *APOA2*, which maximizes mRNA stability [43], TOP-HBB, which maintains the highest ribosome load [43], *mRpl18*, which mediates an intermediate ribosome loading and mRNA stability [43], and *HBA*, which has been used as a 5’UTR for mRNA vaccines [45]. Furthermore, we reasoned that combining two 5’UTRs, one with optimal ribosome loading and the other with enhanced mRNA stability, may further improve translation and in-cell mRNA stability. Thus, we also tested the hybrid *APOA2-TOP-HBB*.

To evaluate the effect of the selected 5’UTRs on protein expression, we inserted the coding sequence of Renilla luciferase (*RLuc*) at the position of the antigens. In the in vitro transcription (IVT) reaction, we used N1-methyl-pseudouridine (m^1^Ψ) instead of uridine, which maximizes protein expression and simultaneously silences mRNA immunogenicity [46–48]. We transfected moDCs with the respective IVT mRNAs containing different 5’UTRs for 24 h. The RLuc expression levels were normalized to those of the Firefly luciferase (FLuc), whose mRNA was co-transfected at an equimolar ratio. Compared to the *HBB* 5’UTR, all 5’UTRs except for *APOA2* significantly enhanced the RLuc expression (Fig. 2B). Notably, *HBA* 5’UTR conferred the highest expression levels, outperforming all other tested 5’UTRs (*P* < 0.0001) (Fig. 2B).

A recent study suggests that (A/U)UUU motifs in m^1^Ψ-modified mRNAs enhance ribosome recruitment, while high G or C content has the opposite effect [49]. We identified a stretch of four cytosines (CCCC) in the 5’UTR *HBA*, which we mutated to AUUU. This alteration did not improve the expression, somewhat decreased it by 3-fold (Fig. S4).

Collectively, these data demonstrate that the 5’ UTR of *HBA* ensures the highest expression and was, therefore, chosen in our mRNA design.

### Optimization of the termination signal to maximize termination fidelity

In addition to the 5’UTR, we also identified the stop codon as a critical parameter to optimize in mRNA vaccines. The replacement of all uridines with m^1^Ψ in the IVT leads to the modification of the stop codons to m^1^ΨAA, m^1^ΨAG or m^1^ΨGA. Ψ-modified stop codon is more susceptible to readthrough [50, 51] through recoding by near-cognate tRNAs [16]. Such recoding events at the stop codon may generate unwanted neoepitopes, which harbor the potential to elicit adverse effects and compromise vaccine safety [52, 53]. The impact of other uridine modifications on termination fidelity is unknown; thus, we considered pseudouridine (Ψ) and 5-methoxyuridine (5moU), which, similar m^1^Ψ, enhance protein expression [54].

To measure stop codon readthrough, we utilized a dual-luciferase reporter mRNA, in which the FLuc reports on readthrough and RLuc is used as an internal control for normalization (Fig. 2C). The test sequence, inserted between RLuc and FLuc, consists of the tested stop codon flanked by 33 base pairs (bp) upstream and 3 bp downstream. The sense GGA codon, encoding glycine, served as a positive control. We tested two different stop codons, UGA and UAA, which previous reports categorize as termination codons with the highest and lowest susceptibility to readthrough, respectively [55, 56]. All different reporter mRNAs were transcribed in vitro by using one of the modified nucleotides. Compared to uridine, modified uridines enhanced the readthrough levels in moDCs irrespective of the stop codon identity (Fig. 2D). At the UGA stop codon, the Ψ modification led to the highest readthrough (3.2%; *P* < 0.0001), consistent with previous reports identifying ΨGA as particularly prone to readthrough [57]. Both m^1^Ψ and 5moU modifications decreased the readthrough level; however, it remained higher than that observed with the unmodified stop codon (Fig. 2D). All modifications also enhanced the readthrough at UAA, however, the overall readthrough was much lower than that at modified UGA codons (Fig. 2D). The nucleoside following the termination codon affects the readthrough, with cytosine enhancing and adenosine reducing the readthrough [58]. Thus, for the UAA stop codon, we tested both variants (UAA(C) and UAA(A)). Changing the nucleotide downstream of the stop codon, from C to A, in combination with any of the modifications, did not significantly affect the readthrough (Fig. 2D).

Given that the UAA stop codon, even when modified, showed lower susceptibility to readthrough, we reasoned that a UAAUAA tandem might further enhance termination fidelity and reduce readthrough. Notably, the tandem UAAUAA stop codons further antagonized the readthrough-stimulating effect of the uridine modifications (Fig. 2D). The effect on the m^1^Ψ was the most pronounced, resulting in a readthrough level even lower than that of the unmodified mRNA (Fig. 2D). The readthrough of the unmodified reporter was similar to that of the reporter with a single UAA (Fig. 2D).

Stop codon readthrough has also been shown to increase the susceptibility of the gene product to lysosomal degradation [59], an effect that would result in lower amounts of the produced antigens and reduced epitope presentation and consequently lower vaccine efficacy. Thus, we also evaluated the protein production in moDCs using the full-length mRNA Rluc reporter (Fig. 2A), which contains a single UAA or a tandem UAAUAA termination signal. mRNAs were IVT-produced using m^1^Ψ. The mRNA with the tandem stop resulted in significantly higher RLuc levels compared to the expression of the mRNA with a single stop codon. The effect of the downstream nucleotide was not significant. Both UAA(C) and UAA(A) showed similar expression levels (Fig. 2E). The UGA(C) stop codon resulted in the lowest expression (Fig. 2E), suggesting that leaky termination decreases the yields of the translated protein.

Together, these results propose that the tandem UAAUAA stop signal fully antagonized the mRNA modification-associated readthrough at stop codons. By minimizing the readthrough, the protein production was enhanced, highlighting the potential safety benefit of incorporating a tandem stop in ex vivo mRNA vaccine design.

### m^1^Ψ modification affects the expression yields and maturation of moDCs

**Fig. 3:**
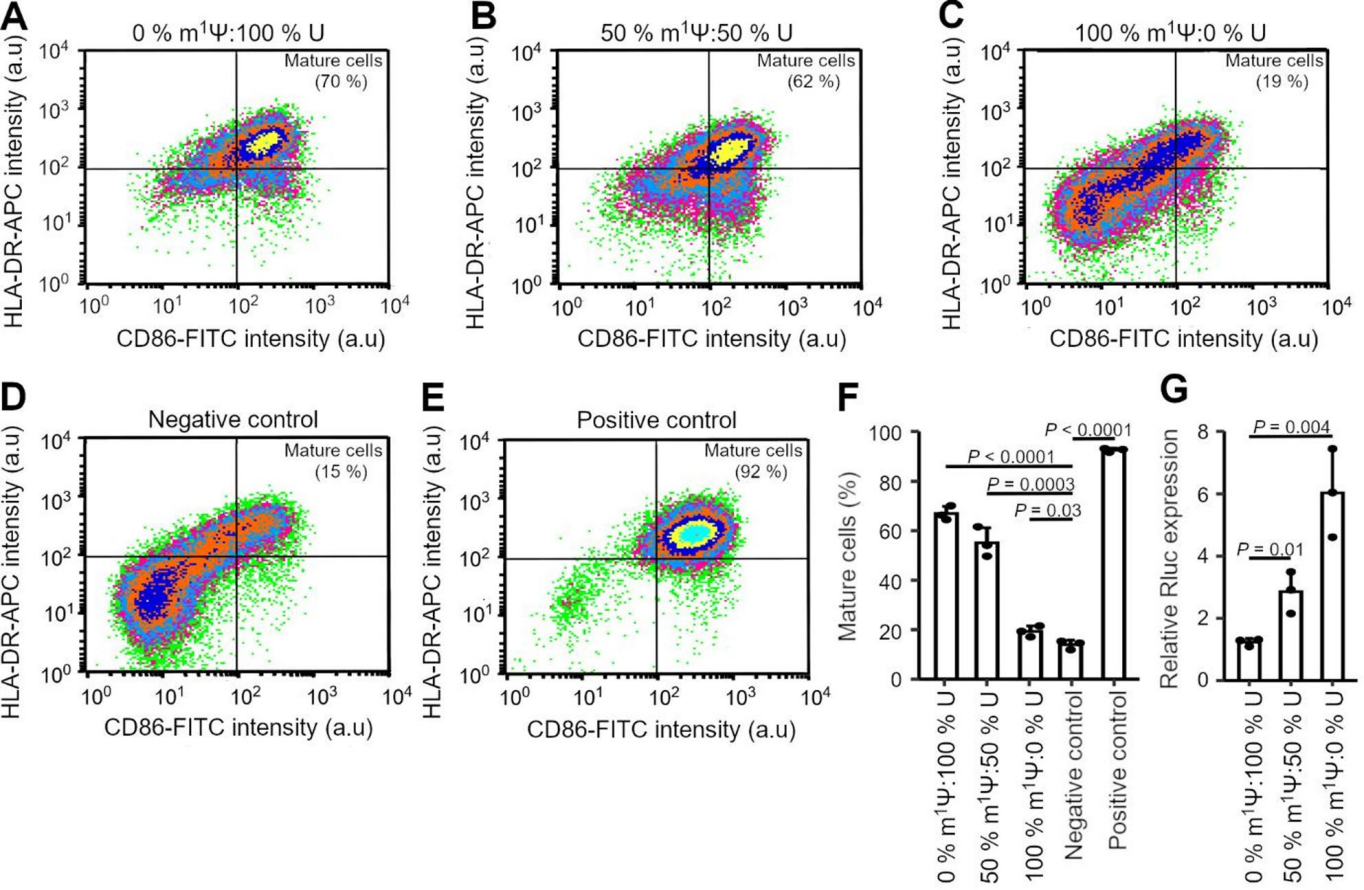
m^1^Ψ modification reduces moDC maturation. **A-E** Representative flow cytometry analysis of the moDC maturation following transfection (24 h) with IVT mRNA containing different m^1^Ψ: U ratios using fluorophore-coupled antibody to detect surface expression of CD86 and HLA-DR. Treatment with the cytokine maturation cocktail served as a positive control, yielding a maturation rate of 92% (**E**). Thresholds for the respective quadrants were determined based on the distribution of cells in the positive control. Cells in the upper right quadrant were classified as mature cells. The percentage of each representative flow cytometry run is in the top right quadrant. **F** Statistical analysis of moDC maturation from panels **A-E**. **G** Relative expression of RLuc reporter normalized to FLuc expression from the IVT mRNAs used in panels A-E. (**F**, **G**) Data are means ± SD (*n* = 3 biological replicates of the FACS analysis). P values were calculated by a two-tailed, unpaired t-test

A recent study suggests that unmodified nucleotides may be beneficial for the design of circulating vaccines, as they enhance the maturation of antigen-presenting cells, thereby increasing the likelihood of epitope presentation [15]. This raised the question as to whether the unmodified mRNA would be more effective as an ex vivo vaccine by enhancing maturation in moDCs. To explore this, we produced mRNAs with different m^1^Ψ:U ratios by adding different ratios between m^1^Ψ and U in the IVT reaction (i.e. 100%:0%, 50%:50%, and 0%:100%) and evaluated the moDCs maturation by flow cytometry using two maturation markers, CD86 and HLA-DR (Fig. 3A-E, Fig. S4). As a benchmark for moDC maturation, we used a cocktail containing interleukin 1β (IL-1β), interleukin 6 (IL-6), tumor necrosis factor α (TNF-α) and prostaglandin E2 (PGE2) [60]. Only moDCs with CD86 and HLA-DR levels comparable to cells treated with the maturation cocktail were classified as mature (Fig. S5). Flow cytometry analysis revealed shifts in the cell distribution dependent on the m^1^Ψ content of the mRNA (Fig. 3A-E). Unmodified mRNA induced the highest moDC maturation (67%) (Fig. 3A, F), whereas with completely m^1^Ψ-modified mRNA, the maturation level dropped to 19% (Fig. 3C, F). These findings suggest that unmodified uridines in mRNA (or a high proportion thereof) promote the enhanced maturation of moDCs in vitro.

While unmodified mRNA induced higher moDC maturation levels, it may negatively affect vaccine efficacy by decreasing protein expression, consequently decreasing the likelihood of epitope processing and presentation. Thus, we transfected moDCs with *Rluc-*encoding mRNA (Fig. 2A), in vitro transcribed with varying m^1^Ψ:U ratios. The level of m^1^Ψ-modification markedly affected the RLuc expression (Fig. 3G). Compared to unmodified mRNA, the m^1^Ψ-modified mRNA displayed a 4.9-fold increase in protein expression (*P* = 0.004) (Fig. 3G).

In an established model system, HEK293 cells stably transformed with TLR7 or TLR8, both of which augmented by single- and double-stranded synthetic and viral RNA [46, 61], the m^1^Ψ-modified mRNA only marginally stimulated TLR7 and TLR8 over the mock transfection control (Fig. S6A, B), thus corroborating the reported low intrinsic immunogenicity of fully m^1^Ψ-modified mRNA [62, 63]. The stimulation in moDCs was monitored by the release of interferon-alpha (IFN-α) and TNF-α, both of which were produced in response to TLR activation [64]. Similar to HEK293 TLR7/TLR8 cells, the m1Ψ-modified mRNA did not significantly activate the innate immune response and release TNF-α and IFN-α (Fig. S6C, D).

Together, these findings imply that the m¹Ψ modification entails a trade-off between moDC maturation and protein expression. Thus, for ex vivo vaccine applications, both parameters should be co-optimized to identify the optimal m¹Ψ:U ratio. Alternatively, fully modified mRNA could be used to maximize protein production, and, in combination with external maturation factors, to enhance moDC maturation, potentially offering a more effective strategy.

### Engineered mRNA vaccine presents epitopes at the cell surface and activates T cells

**Fig. 4:**
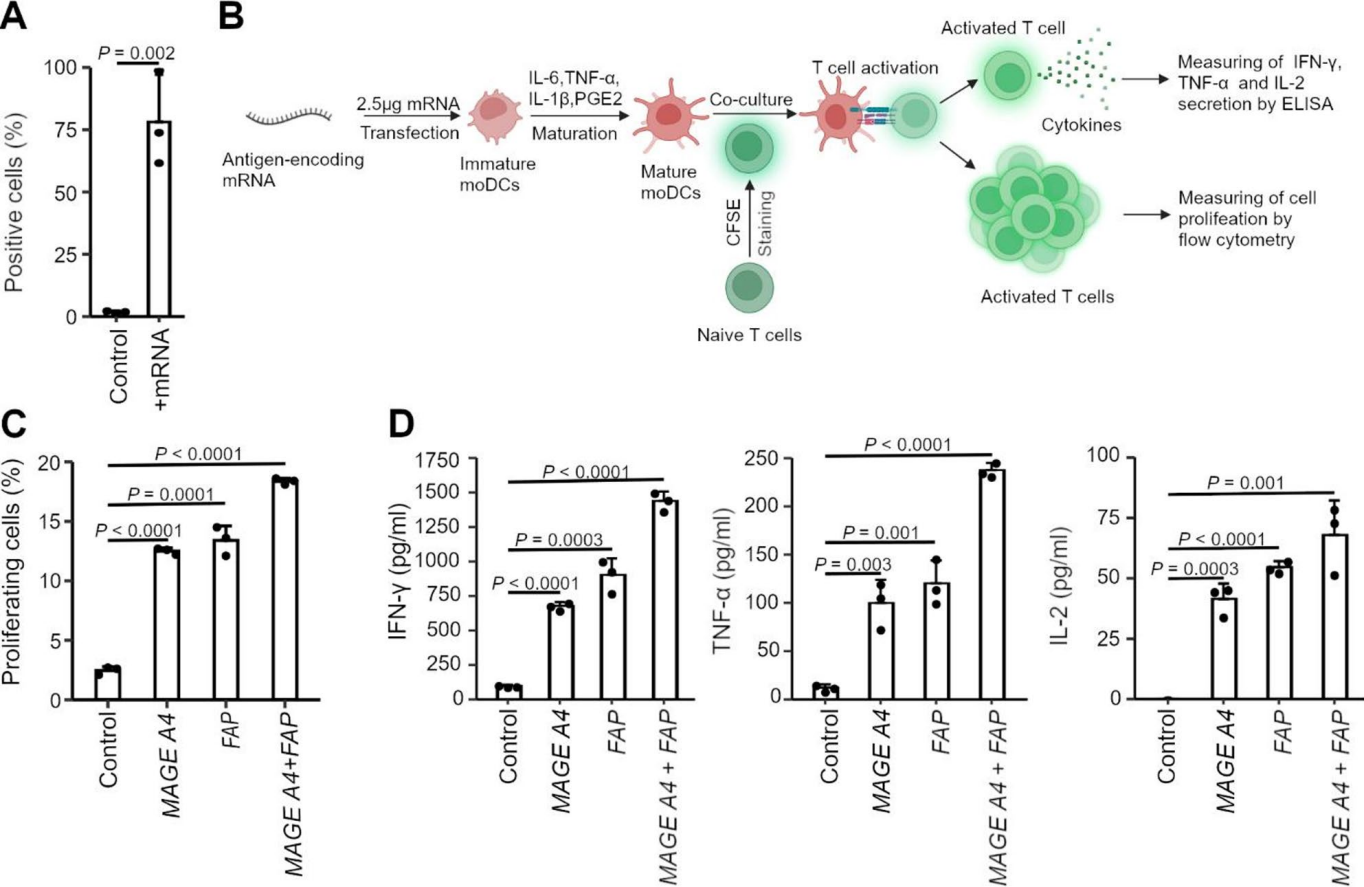
Engineered mRNA facilitates epitope presentation on the cell surface and activates T cells. **A** Statistical evaluation of the flow cytometry analysis of SIINFEKL-positive EL4 cells. Analyses were performed 24 h post-transfection with m^1^Ψ-modfied IVT mRNA encoding SIINFEKL. Control, mock control cells treated with transfection reagent (lipofectamine) only. **B** Schematic of the experimental approach for assessment of T cell activation. **C** Statistical analysis of the flow cytometry analysis for proliferating T cells after 5 days of co-culture with moDCS. **D** ELISA analysis of IFN-γ, TNF-α and IL-2, released into the culture media of T cells and moDCs co-cultured for 5 days after transfection with m1Ψ-modfied IVT MAGEA4 or FAP mRNA. For **C** and **D**, the combinational treatment reflects an equal concentration of the individual mRNAs (1.25 µg each). Control, mock control cells treated with transfection reagent (lipofectamine) only. In all panels, the data are means ± SD (*n* = 3 biological replicates). P values were calculated by a two-tailed, unpaired t-test

Next, we assessed the potential for application as an ex vivo mRNA vaccine by evaluating its ability to produce epitopes and present them on the cell surface, a process crucial for eliciting an effective immune response. To bypass the diversity of human MHC allele combinations and the resulting variability in binding comparisons using human-derived cells, we employed a well-established model system: a peptide fragment of ovalbumin (OVA), SIINFEKL, which binds to the murine MHC class I surface receptor H-2Kᵇ of the mouse-derived T lymphoblast cell line EL-4 [65]. Consequently, we transfected EL-4 cells with *SIINFEKL*-encoding IVT mRNA and assessed peptide-MHC surface presentation by flow cytometry using fluorophore-conjugated antibody binding. In 78% of the cells, transfected with the *SIINFEKL*-encoding IVT mRNA, we observed a marked increase in SIINFEKL presentation on the cell surface 24 h post transfection, compared with 1.7% in the control group transfected with lipofectamine alone (*P* = 0.002) (Fig. 4A, Fig. S7A, B), indicating successful antigen expression, epitope processing and surface presentation, further supporting the potential of the mRNA cassette to be used for ex vivo mRNA vaccination.

### *MAGEA4* and *FAP* expression markedly increase CD8 + T cell proliferation

For an ex vivo mRNA vaccine to elicit a robust immune response, the encoded antigen must successfully activate T cells, particularly CD8 + T cells – the key mediators of cytotoxic response [66]. Thus, we next tested the immunogenic potential of the selected antigens, MAGEA4 and FAP, in the context of the optimized mRNA cassette. To assess their ability to elicit a T cell response, we used a co-culture of moDCs transfected with antigen-encoding IVT mRNA or mock-transfected, and CD8 + T cells isolated from the same human donor (Fig. 4B). Expression of MAGEA4 and FAP, individually or in combination, significantly increased CD8⁺ T cell proliferation compared with the mock control transfected with lipofectamine only (Fig. 4C, Fig. S7C, D), indicating successful antigen expression, epitope presentation, and T cell activation. Notably, co-expression of MAGEA4 and FAP induced greater T cell proliferation than either antigen alone, suggesting the potential benefit of using antigen combinations in the vaccine setting.

To further validate the immunogenic potential of the selected antigens, we measured the release of three key cytokines (i.e. interferon-γ (IFN-γ), interleukin-2 (IL-2), and TNF-α) into the culture medium following T cell activation. Robust secretion of all three cytokines was observed in co-cultures of CD8⁺ T cells and mature moDCs transfected with antigen-encoding IVT mRNA compared to mock-transfected controls (Fig. 4D). IFN-γ level was the highest, consistent with its central role in T cell-mediated immune response [67–69]. In line with the proliferation data (Fig. 4C), the combination of MAGEA4 and FAP induced the strongest cytokine release across all three markers (Fig. 4D).

Collectively, these findings demonstrate that MAGEA4 and FAP, when expressed in the optimized mRNA cassette, effectively elicit a T cell response, particularly when used in combination, highlighting their potential for use in an ex vivo mRNA vaccine for EC.

## Discussion

Here, we report the development of an mRNA-based ex vivo vaccine that addresses the need for more efficient treatment of advanced-stage EC. Using a multi-step selection process and analysis of patient tumor tissue, we identify two antigens, MAGEA4 and FAP, as potential targets for an mRNA vaccine. Functionally, the ex vivo mRNA vaccine elicited a potent T cell response, particularly when combined with both FAP and MAGEA4 antigens, thereby achieving a critical benchmark for further preclinical testing.

 MAGEA4 is a part of the melanoma-associated antigen A (MAGEA) family. It represents a prototypical cancer/testis antigen that is essentially absent in adult somatic tissues and its expression is restricted to tumor cells and immune-privileged germline tissues [70]. Consistently, in EC samples, we found that MAGEA4 protein is exclusively localized in cancer cells and that its expression inversely correlates with disease outcome in EC. Overall, the expression of *MAGE A* proteins is associated with poor survival, resistance to chemotherapy, and metastasis [71–74], making them suitable targets for immunotherapy. Particularly, MAGEA4 has been associated with poor prognosis in non-small cell lung cancer [75]. Several ongoing clinical trials for cancer treatment, including those targeting melanoma and neuroblastoma, utilize MAGEA1 or MAGEA3 as targets [76–79]. Immunotherapy targeting MAGEA4 has gained attention, with a primary focus on adoptive cell therapy for solid tumors, such as lung, ovarian, and urothelial tumors [80, 81].

 FAP is upregulated in tumor cells and cancer-associated fibroblasts (CAFs) within the tumor stroma, but is not expressed in adult somatic tissue [82, 83]. Although upregulation of FAP is frequently associated with reduced OS and increased metastatic potential [84, 85], expression of FAP in specific tumor types, such as breast and pancreatic cancer, has been associated with improved DSF and OS [86, 87], consistent with our findings that FAP expression did not influence OS, but had a positive impact on DSF in EC patients. CAFs in the tumor microenvironment play an essential role in immune evasion by modulating the extracellular matrix and secreting immunosuppressive factors, impairing lymphocyte infiltration to the tumor site [88, 89]. Targeting FAP could disrupt the tumor-promoting stroma and enhance immune infiltration and antigen presentation within the tumor. Consistent with this idea, several studies in murine cancer models demonstrate that targeting FAP and consequent depletion of CAFs, enhances anti-tumor activity and lymphocyte infiltration [90–92]. The combinational approach of co-targeting a tumor cell antigen (MAGEA4) and a CAF antigen (FAP) may enhance and broaden the anti-tumor immune response, potentially overcoming issues of tumor heterogeneity and antigen loss. By simultaneously breaking down stromal immune barriers and inducing tumor cell lysis, the dual-antigen vaccine may create a positive feedback loop of antigen spreading and T cell recruitment, thereby broadening the therapeutic impact on endometrial tumors.

Along with the identification of MAGEA4 and FAP as candidate antigens, which, when combined, effectively stimulate the T cell response, another central advancement of this study is the optimization of the mRNA cassette to enhance antigen expression and ensure higher safety of the vaccine. The α-globin 5′UTR exhibited the most potent effect on the expression output, thus outperforming other common 5′UTRs. Using fully m^1^Ψ-modified mRNA enhances protein yield and also stabilizes mRNA, likely through stimulating stronger base pairing and stacking interactions that stabilize secondary structures [93]. The relatively short 5’UTR of α-globin (e.g. 37 nt compared to the median 5’UTR length of approximately 210 nt in humans) [94] may antagonize the higher propensity of secondary structure formation and thus enhance ribosomal loading onto mRNA stimulating translation and protein production.

Our findings further highlight that modified uridines, widely employed in therapeutic mRNAs, substantially enhance stop-codon readthrough in a manner dependent on stop codon identity (i.e. with UGA(C) exhibiting the highest and UAA(C) the lowest readthrough levels, respectively). The tandem UAAUAA stop in the fully m¹Ψ-modified mRNA serves as an optimal termination signal, minimizing aberrant readthrough and the potential generation of immunogenic neoepitopes.

This proof-of-concept work provides the first evidence that FAP and MAGEA4 antigens delivered via mRNA can drive effective T cell activation. Given that ECs, particularly those with high mutational burden or microsatellite instability, are considered reasonably immunogenic and responsive to immune interventions [6, 7], this mRNA vaccine platform could leverage this immunogenic potential to improve clinical outcomes – a promising step toward more effective immunotherapy-based treatment for EC patients.

## Supplementary Information

Below is the link to the electronic supplementary material.

Supplementary Material 1

## Data Availability

All data included in this study are available upon request by contacting the corresponding authors.
